# Non-destructive Phenotypic Analysis of Early Stage Tree Seedling Growth Using an Automated Stereovision Imaging Method

**DOI:** 10.3389/fpls.2016.01644

**Published:** 2016-10-28

**Authors:** Antonio Montagnoli, Mattia Terzaghi, Nicoletta Fulgaro, Borys Stoew, Jan Wipenmyr, Dag Ilver, Cristina Rusu, Gabriella S. Scippa, Donato Chiatante

**Affiliations:** ^1^Laboratory of Environmental and Applied Botany, Department of Biotechnology and Life Science, University of InsubriaVarese, Italy; ^2^Sensor Systems Department, Acreo Swedish ICTGothenburg, Sweden; ^3^Department of Biosciences and Territory, University of MolisePesche, Italy

**Keywords:** plant phenotype, biomass, seedlings, *Picea abies* L., *Pinus sylvestris* L., *Fagus sylvatica* L., *Quercus ilex* L., RGB image analysis

## Abstract

A plant phenotyping approach was applied to evaluate growth rate of containerized tree seedlings during the precultivation phase following seed germination. A simple and affordable stereo optical system was used to collect stereoscopic red–green–blue (RGB) images of seedlings at regular intervals of time. Comparative analysis of these images by means of a newly developed software enabled us to calculate (a) the increments of seedlings height and (b) the percentage greenness of seedling leaves. Comparison of these parameters with destructive biomass measurements showed that the height traits can be used to estimate seedling growth for needle-leaved plant species whereas the greenness trait can be used for broad-leaved plant species. Despite the need to adjust for plant type, growth stage and light conditions this new, cheap, rapid, and sustainable phenotyping approach can be used to study large-scale phenome variations due to genome variability and interaction with environmental factors.

## Introduction

Worldwide, an estimated two billion ha of forests are degraded (Minnemayer et al., 2011; Stanturf et al., 2014). In addition to the continuing anthropogenic alterations of global ecosystems (Foley et al., 2005; Kareiva et al., 2007; Ellis et al., 2013), the anticipated effects of global climate change are expected to lead to further deforestation and forest degradation in the future (Steffen et al., 2007; Malhi et al., 2008; Zalasiewicz et al., 2010; Stanturf et al., 2014). Recently, restoration of degraded land has received increasing attention due to its potential to reconcile agricultural development and forest conservation (Robertson and Swinton, 2005; Kissinger et al., 2012). Among the many techniques and tools available for restoration strategies (Stanturf et al., 2014), container seedlings may be the most cost-effective when the planting season is to be extended or adverse sites are to be planted (Brissette et al., 1991; Luoranen et al., 2005, 2006; Stanturf et al., 2014). Container seedlings are produced to meet desired characteristics for outplanting under specified conditions (Brissette et al., 1991; Landis et al., 2010). This requires the artificial production of high-quality forest planting stock material (Wang et al., 2007; Cole et al., 2011) able to successfully survive and grow after outplanting (Wilson and Jacobs, 2006). To achieve this, there is an urgent need to improve the phenotypic assessment of containerised tree seedlings.

The phenotype of a plant is the result of a complex interaction between morphological, ontogenetical, physiological, and biochemical factors (Gratani, 2014). A thorough knowledge of the phenotypic variation occurring spontaneously in nature or after induction by non-intrinsic factors such as environmental stressors is essential for a better understanding of all events taking place in the life of a plant (Grant-Downton and Dickinson, 2006; Kuromori et al., 2009). For the purposes of this paper, we refer to phenotyping as a method to measure plant growth using non-invasive technologies that have become increasingly available in recent years (Fiorani and Schurr, 2013). Unfortunately, measurements of relative growth rate on a mass basis still depend on destructive and time-consuming approaches (Walter et al., 2007; Fiorani and Schurr, 2013; Humplík et al., 2015) with the result of limiting the possibility to examine (1) a large number of samples enabling metadata analysis, and (2) the same sample repeatedly over time (Furbank and Tester, 2011; Busemeyer et al., 2013; Rahaman et al., 2015). To overcome these constraints and to increase the usefulness of phenotype investigation, new approaches based upon the use of technologically advanced equipment that do not affect the samples under examination have been attempted (Tsaftaris and Noutsos, 2009; Walter et al., 2015). Among these, the one based on a non-destructive image analysis seems to achieve a good reliability for rapid phenotyping measurements of a number of plant traits (Li et al., 2014; Humplík et al., 2015). The reliability of this approach was demonstrated in shoot growth rate analyses in which increments measured as differences of digital area showed a high degree of correlation with those obtained by traditional fresh or dry weights measurements (Humplík et al., 2015; Rahaman et al., 2015; and reference herein). A further improvement of this approach is likely to contribute to a better understanding of the principles governing plant biomass distribution in all organs during the lifespan of a plant, a factor of primary importance for phenotype determination. Similarly, other investigations based on measurements of morphometric parameters (i.e., leaf area, stem height, number of tillers, and inflorescence architecture) of plant growth in controlled and natural conditions could benefit from adopting this non-destructive approach (Busemeyer et al., 2013; Fiorani and Schurr, 2013; Rahaman et al., 2015). Moreover, in recent years a large body of literature is rapidly accumulating, mainly for *Arabidopsis* and agricultural plant species, demonstrating how non-destructive analysis of plant phenotype supports other omics approaches to plant science (Edwards and Batley, 2004; Kuromori et al., 2009). However, despite the undeniable merits of this non-destructive method, it cannot be ignored that a number of biases affect these measurements due to overlapping, twisting, curling, and circadian movement of plant organs during image acquisition, especially when 2D color red–green–blue (RGB) image is taken from a single direction (top view) (Lati et al., 2013; Tessmer et al., 2013; Humplík et al., 2015). Indeed, it is difficult to reliably separate overlapped plant canopies into individual plants and the development and implementation of these methods is limited to early growth stages of a specific plant (Jin and Tang, 2009). To overcome these biases the utilization of a stereo vision system has advantages over conventional 2D machine vision-based plant sensing systems (Jin and Tang, 2009; Piron et al., 2009; Lati et al., 2013). Even though stereo vision system appear promising for estimation of plant growth parameters and development of models, development and implementation of these methods is still limited in terms of species and plant developmental stage (Lati et al., 2013).

We have specifically developed a simple and flexible optical system together with its associated imaging and processing software able to compare acquired images and to obtain, rapidly and efficiently, measurements of height and greenness of young containerised seedlings during the precultivation period. Unlike most of the commercially available solutions for plant phenotyping which are costly and require a large space (Granier et al., 2006; Tsaftaris and Noutsos, 2009), our system is low cost and has the dimension of a bench instrument. In particular, the small size characteristic makes the system easily transportable and combinable with other equipment as well as with high potential to be straightforward integrated in mass-industry. In the present paper, we describe this in-house developed optical system together with the results obtained from a growth kinetics study on tree seedlings grown in a growth chamber, from seed germination to 5-weeks-old plants. Plant biomass is defined as the total mass of all the above- and below-ground parts at a given point in a plant’s life (Roberts et al., 1993; Humplík et al., 2015; Wang and Ruan, 2016). The rationale for testing the functioning of our system with this important parameter is twofold: (a) its considerable influence on the plant phenome, and (b) its great variability in response to environmental factors (Coleman et al., 1994; Di Iorio et al., 2011; Montagnoli et al., 2012, 2014; Chiatante et al., 2015). In order to widen the implementation and development of stereo vision method, seedling analysis was performed with four different species characterized by different canopy geometries and development, two broad-leaved (*Fagus sylvatica* L., *Quercus ilex* L.) and two needle-leaved (*Picea abies* L., *Pinus sylvestris* L.). Since different species and types of plants are characterized by differences in architectural organization (Barthélémy and Caraglio, 2007; Díaz et al., 2016), the effectiveness of our in-house built optical system and its corresponding software was characterized by using both broad-leaved and needle-leaved. We present also a comparison between the data obtained by automated imaging analysis with those obtained with the traditional destructive method.

## Materials and Methods

### Plant Material and Growth Chamber Characteristics

Seeds of four tree species (*Fagus sylvatica* L., *Quercus ilex* L., *Picea abies* L., and *Pinus sylvestris* L.) were provided by the National Forest Service (National Centre for Study and Conservation of Forest Biodiversity-Peri, Italy) and sorted for uniform size. Seeds of *F. sylvatica* were first hydrated by soaking for 24 h in tap water; then seeds were surface sterilized with 3,5% household bleach for 2 min, and rinsed four times with sterile water to remove all traces of bleach. Afterward, seeds were treated with “Teldor” fungicide (3 ml in 1 l of sterile water per 10 min) and placed under a hood for 3 h to improve fungicide adherence to the seed coat. Finally, seeds were subjected to cold stratification in perlite at 4°C for 2 months. Seeds of *Q. ilex* were hydrated by soaking them for 24 h in tap water and sown without further pretreatment. *P. sylvestris* and *P. abies* seeds were directly sown directly without any pretreatment. A total of 104 seeds were sown in four different mini-plug plastic container trays (QPD 104 VW – 104 cells; 33 mm × 33 mm × 45 mm; 40 mm/height; 27 cc) (QuickPot by HerkuPlast-Kubern, Germany), containing sterile stabilized peat growing medium Preforma VECO3 (Jiffy^®^ Products). The temperature and humidity settings in the growth chamber are detailed in **Table 1**. The trays were placed on a steel table with a 50 mm-high edge in order to fill it up with water. The mini-plugs had drainage holes in their base, allowing watering from underneath. Watering operations were made every 3 days during germination and every 2 days during growth period to maintain constant water content in each tray. Seed germination was 78% for *Q. ilex*, 66% for *F. sylvatica*, 78% for *P. sylvestris*, and 96% for *P. abies*. Plants were grown under fluorescent light (FLUORA T8), yielding approximately 120 μmolm^-2^ s^-1^ (Light Meter sensor – HD2302.0 – Delta Ohm, Italy) at tray height. Each plant species was grown independently in the same chamber until the harvest date. A single growth chamber was used to allow for a strict control of environmental factors (uniform conditions) and seedling development (coetaneous cohort).

**Table 1:** Growth chamber settings (number of dark/light hours, relative temperatures, and humidity) for each species.

Plant species	Photoperiod (h) day/night	Temperature (°C)	Relative humidity (%)
*Pinus sylvestris* L.	16/8	21–26	80 (germination),
			55–70 (growth)
*Picea abies* L.	16/8	21–26	80 (germination),
			55–70 (growth)
*Fagus sylvatica* L.	16/8	21–22	70
*Quercus ilex* L.	16/8	21–22	70

### Experimental Design

For each species, four trays were grown for a total of 416 seedlings (104 seedlings per tray). To investigate the kinetics of plant growth, half a tray was considered for destructive analysis and the other half for non-destructive image analysis. The first sampling point was 14, 15, and 21 days after germination (a.g.) depending on the plant species. Following samplings were carried out at intervals of no less than 6 days and not more than 12 days depending on the plant species, for four sampling points and 4 weeks of growth period.

### Measurement of Shoot Height and Plant Biomass

At each sampling date, plant height of seedlings for non-destructive analysis (*n* = 52) was measured manually with a wooden measuring stick from the base of the seedling to the highest leaf. Furthermore, five seedlings per tray (20 seedlings in total per species) were randomly collected at each sampling point. Leaves, shoots, and roots from each seedling were oven dried (52 h at 75°C) and weighed in order to measure total plant biomass.

### Optical System

The optical data acquisition system consists of two digital color cameras equipped with identical lenses from Edmund Optics: 1/1.8” CMOS, 1280 × 1024 pixels, sensor area 6.79 × 5.43 mm, 5 mm fixed focal length lens, field-of-view of 65.5° (UI-1240SE: USB 2.0 uEye industrial camera from IDS Imaging^1^). A rugged USB cable is used for both data transmission and supplying the current to the camera electronics.

The cameras are mounted next to each other as close as possible (∼5.5 cm) for stereographic imaging technique for the image color extraction of plant-green and for plant height estimation.

### Image Capture

**FIGURE 1:**
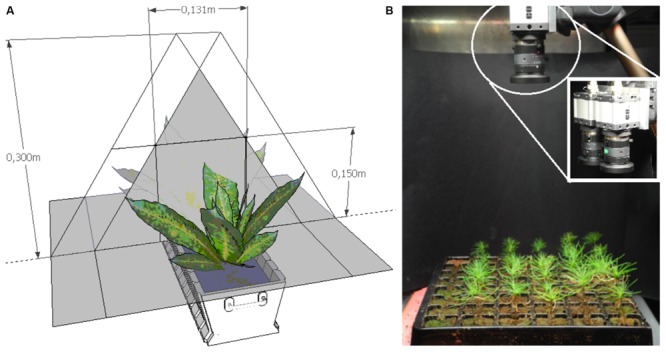
**(A)** Scheme of optical sensing set-up based on stereoscopic measurements. **(B)** Optical system for measuring shoot height and greenness; zoom-in shows the dual cameras for stereoscopic imaging.

Shoot stereoscopic images were taken at the same time as the destructive sampling. The trays were manually moved into the image capture cabinets where one stereoscopic image – top view – of each experimental half tray was taken. The tested optical sensing system is based on image acquisition and data processing using in-house developed algorithms derived from hue-saturation-value (HSV) analysis of the image data. Shoot height sensing is based on analysis of reflected light by using a stereoscopic imaging system (**Figure 1**). Total leaf area or green biomass sensing is based on analysis of reflected light using the percentage of green ground coverage by foliage when observed from above. The same hardware is used for extraction of plant greenness and stereoscopic analysis. The depth of focus of the image is a combination of sensor size, focal length and aperture of the lens, and the distance between camera and object. This system can measure various leave colors (e.g., green, red–brown) and different seedling heights (e.g., 4–5 cm, 15–20 cm). The green pixel selection is sensitive to the light source; the proper configuration is also controlled by the .ini file for the respective camera. In particular, parameters in .ini file such as timing (pixel clock, frame rate, exposure time), master and color gain (red, blue, and green), were adjusted given their effect upon green pixel selection. In fact, by setting these parameters it was possible to level out the image colors recorded by the two cameras as well as to distinguish more clearly leaves within the background frame. A long enough sequence of these images can be used to provide a time-series of plant growth – averaged either over the entire scene, or for individual plants. The achieved resolution of the height map is about 1mm that is adequate to follow plant development. After image capture, all images were analyzed using uEyeDualcam and HeightMap software products (Acreo Swedish ICT).

### Software for Data Acquisition with Optical System

The control of the cameras is carried out using a vendor-supplied software library, uEye (from IDS GmbH). This library is linked to a graphical user interface (GUI) developed in-house in Microsoft Visual C++. From now on, our developed GUI software executable is referred to as uEyeDualCam.

This uEyeDualCam software has been designed to both functioning for the configuration of individual parameters for each camera as well as the extraction of the “green-only” information for each picture taken. In particular, the automatic setup of individual parameters configuration can be performed by means of special initialization (.ini) files for each camera. The .ini files can be edited by hand providing individual setups for the different light sources because, for example, the green-pixel selection is sensitive to the characteristics of the light source. Moreover, the “green-only” information can be extracted and saved in the PNG format, as a picture. The extraction of the pixels with the relevant shades of plant color is accomplished by converting the color information from RGB format into the HSV format. Both formats are commonly used in image processing and the conversion algorithm is free. This step is relatively simple but time-consuming as each image contains approximately 1.3 Mpixels for our system. Afterward, the uEyeDualCam software allow editing the selection of useful HSV color information corresponding to the “green-only,” whatever color characterize leaves of the species analyzed (e.g., bright green, reddish green, or orange). The shades of useful color form a cylindrical segment in the HSV color space. The uEyeDualCam software selects only pixels within that segment, whereby the non-plant pixels are replaced by the black color. At this point, the uEyeDualCam software provides the percentage of plant pixels for the currently processed image.

A separate set of processing tools (HeightMap software) was developed for the function of height-mapping of each stereoscopic image pair. A discussion of the basic principles of stereoscopic analysis is available from Ensenso and Ids Imaging Development Systems GmbH (2012). In particular, the HeightMap software recalculated greenness using “green-only” information in order to create a plant height map (cm) of the tray conferring a value to the pixel of selected images. Thus, the main innovation in our work is the removal of the soil background and keeping only the plant information within each image. This improves the processing speed and the ability of the tool to match/correlate the relevant image pixels without interference. The current revision of the HeightMap software allows for computing the height distribution of each image at the pixel level, within a selectable sub-set of the scene. The HeightMap software provides a pixel map for the entire scene that can be saved in the monochromatic PNG format.

### Statistical Analysis

Morphological measurements were square root or log transformed to ensure normal distributions and equal variances for the use of parametric statistics. Analysis of variance (one-way ANOVA) was carried out to test the effect of time on plant height, greenness, and biomass. *Post hoc* Bonferroni tests were conducted to detect significant differences between sampling days. An independent samples *t*-test was applied to test the significance of differences between plant height obtained by destructive sampling and plant height obtained by sensor analysis for each sampling date. Analyses of parametric methods were applied at a 95% significance level.

Data of plant height and greenness were related to seedling biomass and allometric equations were obtained by regression analysis. Significant equations were used to develop a regression growth model for each species based on the variation of plant height or greenness over time. In order to test the performance of applied models, the relative root mean squared error (RMSE%) and the relative model bias (BIAS%) were calculated by comparing biomass values predicted from plant height or greenness model with actual biomass values in the range of measured values. Statistical analysis was carried out using statistical software package SPSS 17.0 (SPSS Inc, Chicago IL, USA).

## Results and Discussion

### Shoot Height and Plant Biomass

**FIGURE 2:**
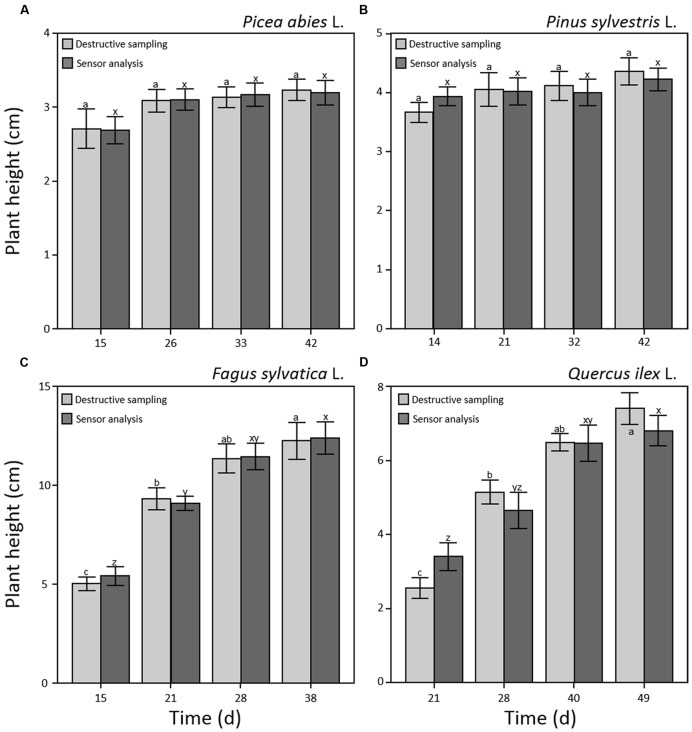
**Plant height (cm) during early seedling development measured by optical sensors (dark gray) and destructive sampling (light gray) for *Picea abies* (A), *Pinus sylvestris* (B), *Fagus sylvatica* (C), and *Quercus ilex* (D).** Data refer to each sampling date after germination and are represented as means (*n* = 52) ± 1 SE. Lowercase letters indicate statistically significant differences (*p* < 0.05) between each sampling date (a, b, and c) and between sensor and destructive analysis (x, y, and z).

**FIGURE 3:**
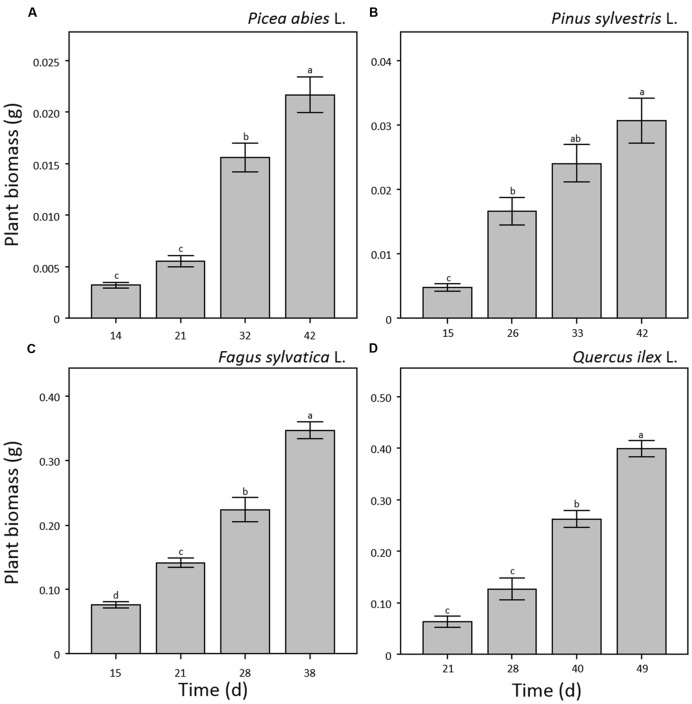
**Plant biomass (g) measured by destructive sampling for seedlings of *Picea abies* (A), *Pinus sylvestris* (B), *Fagus sylvatica* (C), and *Quercus ilex* (D).** Data refer to each sampling date after germination and are represented as means (*n* = 5) ± 1 SE. Lowercase letters indicate statistically significant differences (*p* < 0.05) between each sampling date.

Results on plant height did not show significant differences between manual and software measurements for all four species and sampling points (**Figure 2**) demonstrating that the combination of optical sensors and software analysis constitutes a valuable alternative to destructive methods. Shoot height throughout the experiment showed different patterns for needle- and broad-leaved species (**Figure 2**). In the case of both needle-leaved species, no significant increment of plant height was detected after the emergence of cotyledons (*p* = 0.240 and *p* = 0.256 for *P. abies* and *P. sylvestris*, respectively; **Figures 2A,B**) as internode elongation did not occur during the consecutive emissions of new leaves at this early developmental stage. Therefore, seedlings reached almost maximum height at the first sampling point (day 14th and 15th a.g., respectively), with a slight not significant increment detectable at the last sampling point (day 42nd a.g.; **Figures 2A,B**). Our results fall within the range of the rates measured by other researchers for pine species (Jarvis and Jarvis, 1964; Grime and Hunt, 1975; Grotkopp et al., 2002). Moreover, our findings are in line with those of other authors (Evans, 1972; Causton and Venus, 1981; Hunt, 1982; Grotkopp et al., 2002) who showed that growth of *Pines* typically increases sharply between 2 and 4 weeks after seedling emergence and then declines over time. This is probably due to the invaders habit of *Pinus* species characterized by high growth rate, small seed mass, and short generation time (Grotkopp et al., 2002; de Chantal et al., 2003). Broad-leaved species showed a different growth pattern. Plant height increased significantly (*p* < 0.001) throughout the experiment that reached a maximum value of 13 and 7 cm for *F. sylvatica* and *Q. ilex*, respectively, at the third sampling point (day 28th and 40th a.g.), without further increment until the end of the experiment (**Figures 2C,D**). As plant growth at this stage of seed development is still depending on endogenous factors (Bentsinka and Koornneef, 2008; Baskin and Baskin, 2014), the observed pattern is probably attributable to species-specific growth habits. Despite the importance of early seedling development, studies evaluating this process remain scarce or absent (Walter et al., 2007) as in the case of *F. sylvatica* and *Q. ilex*. Concerning plant biomass development, all four species showed a significant power function increase (*p* < 0.001) throughout the experiment (**Figure 3**). Moreover, the two broad-leaved species (**Figures 3C,D**) showed a 10-fold higher total biomass than needle-leaved species (**Figures 3A,B**).

### Shoot Greenness

**FIGURE 4:**
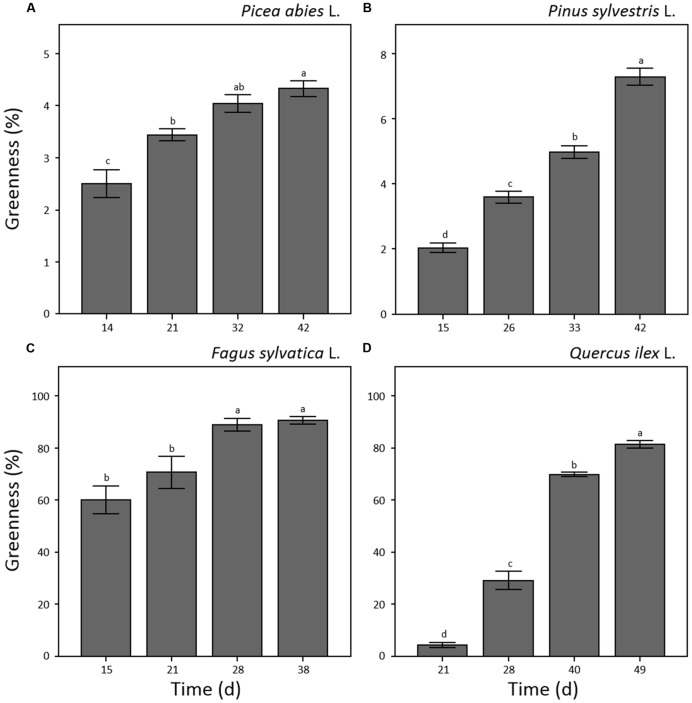
**Plant greenness (%) measured by optical system analysis for seedlings of *Picea abies* (A), *Pinus sylvestris* (B), *Fagus sylvatica* (C) and *Quercus ilex* (D).** Data refer to each sampling date after germination and are represented as means (*n* = 52) ± 1 SE. Lowercase letters indicate statistically significant difference (*p* < 0.05) between each sampling date.

Shoot greenness of the seedlings showed significant variation throughout the experiment (*p* < 0.001) with different patterns for each of the considered species (**Figure 4**). In the case of *F. sylvatica*, the maximum value was reached at the third sampling point (day 28 a.g.; **Figure 4C**) remaining stable until the end of the experiment. *P. abies*, *P. sylvestris*, and *Q. ilex* (**Figures 4A,B,D**) showed a continuous increase in greenness throughout the experiment reaching maximum values at the last sampling point (day 42, 42 and 49 a.g., respectively). In general, broad-leaved species showed 10–20 time fold higher values of greenness than needle-leaved species (**Figures 4C,D**). Seedling leaves of *F. sylvatica* covered almost 80% of the trays at day 21 a.g. while *Q. ilex* reached 80% tray coverage at day 49 a.g. (**Figures 4C,D**). On the other hand, *P. abies* and *P. sylvestris* covered less than 7% of the total tray area at 42 days a.g. (**Figures 4A,B**).

### Regression Model

In order to test our non-destructive measurement method as a tool for monitoring tree seedling growth, patterns of tray greenness and seedling height obtained by software analysis, were related to seedling biomass data obtained by classical destructive analysis. The power function was selected as best fit for all the relationships. This might be explained by Richards (1959) which demonstrated that in the allometric relationship between two correlated growth characteristics, throughout plant development, if the known growth characteristic is conforming to one curve type, any other unknown characteristic increasing allometrically with it will have the same family of growth curve. In our case, plant height and greenness growth characteristics were related to plant biomass which developed in time conforming to power function curve.

**FIGURE 5:**
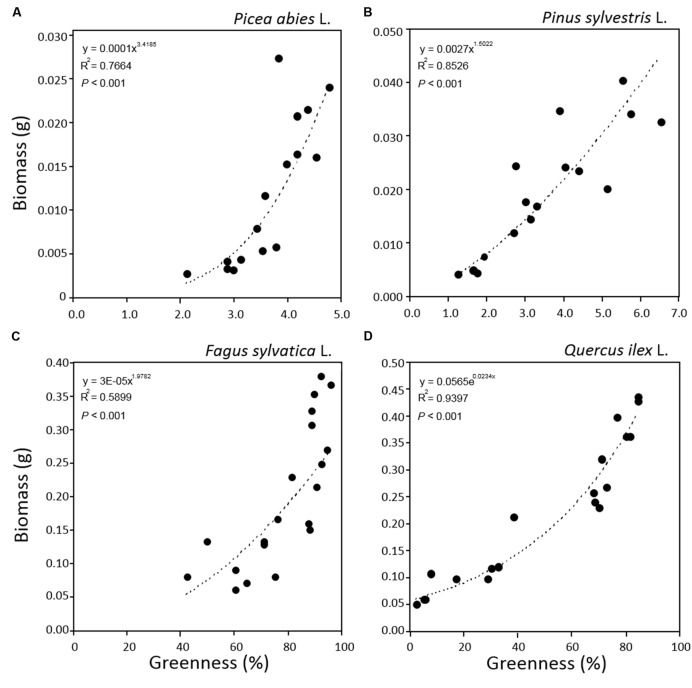
**Relationships between greenness and plant biomass for seedlings of *Picea abies* (A), *Pinus sylvestris* (B), *Fagus sylvatica* (C), and *Quercus ilex* (D).** Data refer to all sampling dates.

**FIGURE 6:**
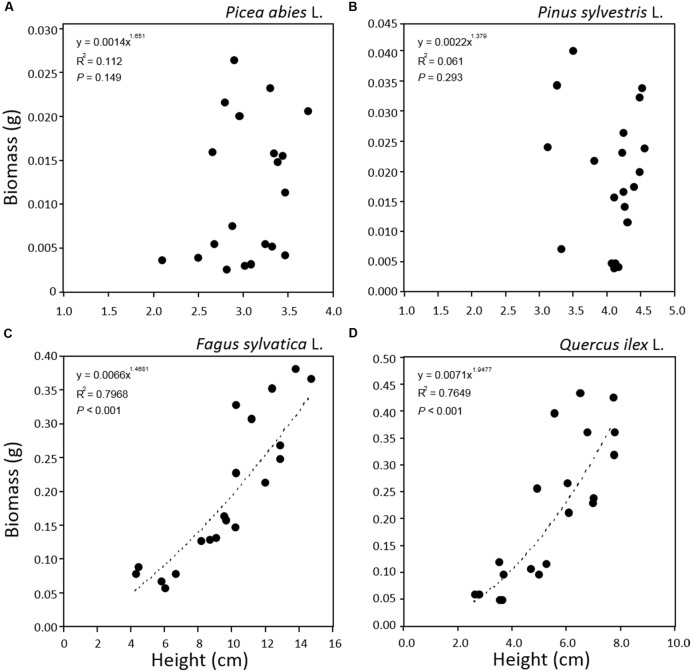
**Relationships between plant height and plant biomass for seedlings of *Picea abies* (A), *Pinus sylvestris* (B), *Fagus sylvatica* (C), and *Quercus ilex* (D).** Data refer to all sampling dates.

**FIGURE 7:**
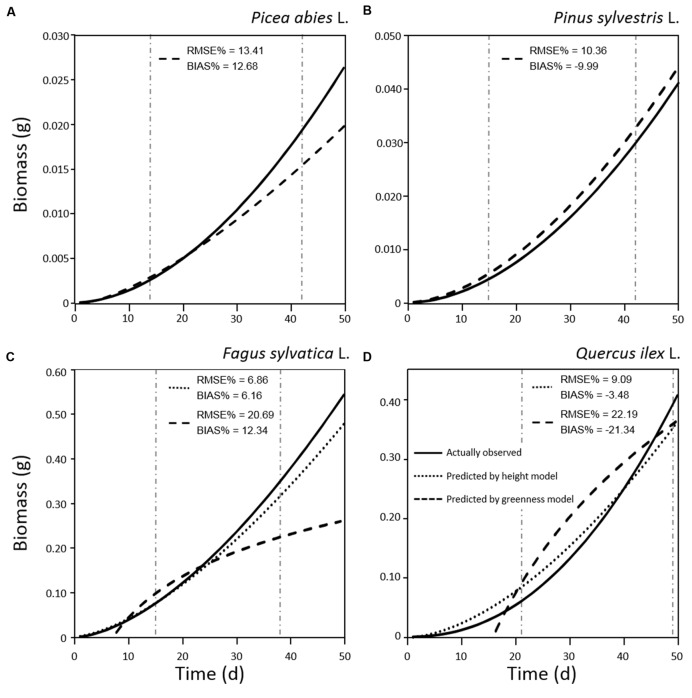
**Regression models of biomass growth for seedlings of *Picea abies* (A), *Pinus sylvestris* (B), *Fagus sylvatica* (C), and *Quercus ilex* (D), calculated by destructive sampling (actually observed; solid line) and by plant height (dotted line) and greenness (dashed line) models.** RMSE% and BIAS% values were calculated comparing values predicted by plant height or greenness models with actual observations in the range of measurements (vertical dot-dashed lines).

The relationship between tray greenness and seedling biomass showed a good correlation for all species until the tray was almost fully covered (**Figures 5**). However, in the case of *F. sylvatica* almost the whole tray was covered in less than 1 month but, as its biomass continued to increase after full coverage (**Figures 3C** and **4C**), a lower coefficient of determination (**Figure 5C**) was observed. This might be due to deviation in the segmented plant’s area occurring when different plant leaves overlap (Jin and Tang, 2009; Lati et al., 2013). Alternative approaches are offered by stereovision-based models which allow plant characterization using 3D spatial properties. A weak relationship between seedling height and biomass was found in the case of the two needle-leaved species (**Figures 6A,B**) while a strong relationship was found for the two broad-leaved species (**Figures 6C,D**). Indeed, both needle-leaved species (*P. abies* L. and *P. sylvestris* L.) did not significantly increase plant height during the growth period (**Figures 2A,B**) despite the continuous increment of seedling biomass. Regression growth models, derived from the relationship of both height and greenness with biomass and their variation with time (**Table 2**), were compared with allometric equations obtained by destructive sampling (**Figure 7**). Therefore, the greenness regression growth model showed the best and the only fit for *P. abies* and *P. sylvestris* (**Figures 7A,B**). Moreover, the RMSE values showed the best fit to the plant height regression growth model for *F. sylvatica* and *Q. ilex* (**Figures 7C,D**). As highlighted by previous studies (Downie et al., 2012; Humplík et al., 2015; Walter et al., 2015), image analysis is a robust method to record three-dimensional information. Our study confirms this, showing a good fit to models with destructive data. Furthermore, in the present work is clearly demonstrated how crucial the choice of the parameter to analyze is, depending on the species under investigation and the growing conditions. In particular, for our system, the possibility to choose plant greenness or plant height parameters enhances the analysis over the other already existing systems. In particular, the results obtained by stereo optical system were highly comparable with results obtained with direct destructive methods. This highlighted how the ‘choose’ function strongly reduced problems often occurring in image-based phenotyping such as overlapping, twisting, curling, and circadian movement. Indeed, in the case of broad-leaved species (*F. sylvatica* and *Q. ilex*) that fully cover a tray, the plant height parameter works better than greenness because new leaves, although related to an increase of plant height, overlap with other leaves and quickly cover the entire tray. On the other hand, the greenness parameter works better than plant height for needle-leaved species (*P. abies* and *P. sylvestris*) because new leaves do not overlap while plant height remains almost constant during the early growth period.

**Table 2:** Regression growth model obtained by actually measured data and by predicted data based on height and greenness measured by optical sensors.

Species	Equation		*R*^2^
*Picea abies* L.	Actually measured	Biomass = 0.00002 × Time^1.8344^	0.93
	Predicted based on greenness	Biomass = 0.00004 × Time^1.5931^	0.77
*Pinus sylvestris* L.	Actually measured	Biomass = 0.00003 × Time^1.8447^	0.88
	Predicted based on greenness	Biomass = 0.00004 × Time^1.7889^	0.93
*Fagus sylvatica* L.	Actually measured	Biomass = 0.0009 × Time^1.6366^	0.95
	Predicted based on greenness	Biomass = 0.1384 × ln(Time) – 0.2741	0.67
	Predicted based on plant height	Biomass = 0.0012 × Time^1.5317^	0.77
*Quercus ilex* L.	Actually measured	Biomass = 0.00007 × Time^2.2141^	0.92
	Predicted based on greenness	Biomass = 0.3118 × ln(Time) – 0.884	0.95
	Predicted based on plant height	Biomass = 0.0005 × Time^1.6824^	0.69

*Models estimate biomass values at a certain time (day).*

## Conclusion

Automatic plant phenotyping is under rapid development due to its potential for comparative phenotyping of a large number of samples in an easy, rapid and not-destructive manner. Therefore, technological effort is being put into the development of a low cost and more accessible phenotyping stereo vision system. Here we present a simple and flexible system that is less inexpensive compared to most of the solutions available today on the market and does not require any specific skills to be run. The data collected refer to a comparative analysis of a number of morphological traits obtained from containerised tree seedlings at their precultivation stage. Results suggest that at present the system is reliable, allowing for straightforward control and adjustment of various plant and light source parameters. Therefore, the system and models developed provided a strong agreement between the actual and estimated growth parameters for plants with interconnected canopies. Although the possibility to adapt the system to other growing conditions, conclusion of the present work are specie-specific and focus on containerized early stage of seedlings. Further implementation in both software and hardware can be done for improving the characterization efficiency of bigger plants, different species and light conditions. Finally, the phenotyping approach used to measure the growth of young seedlings it might also be of support to different omics investigations.

## Author Contributions

AM make substantial contributions to the study concept and design, to data collection process and relative interpretation. AM writes the article and dealt with manuscript process, improvements and revisions. MT participate to all works aspects such as concept and design, lab work, software development, data collection and analysis. NF contribute to the experiment design, seedlings growth and data collection. BS, JW, DI, and CR make substantial contribution to the software development and hardware settings for image acquisition and analysis. BS and CR equally contribute in drafting and revising the article concerning the optical sensors and image data acquisition parts. GS supervise the research and contribute to all works aspects. DC conceive and supervise the research in all aspects. Give important intellectual content in outlining the article and revising it critically.

## Conflict of Interest Statement

The authors declare that the research was conducted in the absence of any commercial or financial relationships that could be construed as a potential conflict of interest.

## Footnotes

1 https://en.ids-imaging.com/store/ui-1240se.html
